# Nitric Oxide Accumulation: The Evolutionary Trigger for Phytopathogenesis

**DOI:** 10.3389/fmicb.2017.01947

**Published:** 2017-10-10

**Authors:** Margarida M. Santana, Juan M. Gonzalez, Cristina Cruz

**Affiliations:** ^1^Centro de Ecologia, Evolução e Alterações Ambientais (cE3c), Faculdade de Ciências, Universidade de Lisboa, Lisboa, Portugal; ^2^Instituto de Recursos Naturales y Agrobiología, Consejo Superior de Investigaciones Científicas (CSIC), Sevilla, Spain

**Keywords:** denitrification, aerobic respiration, horizontal gene transfer, nitrite reductase NirS, *Thermus thermophilus*

## Abstract

Many publications highlight the importance of nitric oxide (NO) in plant–bacteria interactions, either in the promotion of health and plant growth or in pathogenesis. However, the role of NO in the signaling between bacteria and plants and in the fate of their interaction, as well as the reconstruction of their interactive evolution, remains largely unknown. Despite the complexity of the evolution of life on Earth, we explore the hypothesis that denitrification and aerobic respiration were responsible for local NO accumulation, which triggered primordial antagonistic biotic interactions, namely the first phytopathogenic interactions. N-oxides, including NO, could globally accumulate via lightning synthesis in the early anoxic ocean and constitute pools for the evolution of denitrification, considered an early step of the biological nitrogen cycle. Interestingly, a common evolution may be proposed for components of denitrification and aerobic respiration pathways, namely for NO and oxygen reductases, a theory compatible with the presence of low amounts of oxygen before the great oxygenation event (GOE), which was generated by Cyanobacteria. During GOE, the increase in oxygen caused the decrease of Earth’s temperature and the consequent increase of oxygen dissolution and availability, making aerobic respiration an increasingly dominant trait of the expanding mesophilic lifestyle. Horizontal gene transfer was certainly important in the joint expansion of mesophily and aerobic respiration. First denitrification steps lead to NO formation through nitrite reductase activity, and NO may further accumulate when oxygen binds NO reductase, resulting in denitrification blockage. The consequent transient NO surplus in an oxic niche could have been a key factor for a successful outcome of an early denitrifying prokaryote able to scavenge oxygen by NO/oxygen reductase or by an independent heterotrophic aerobic respiration pathway. In fact, NO surplus could result in toxicity causing “the first disease” in oxygen-producing Cyanobacteria. We inspected in bacteria the presence of sequences similar to the NO-producing nitrite reductase *nirS* gene of *Thermus thermophilus*, an extreme thermophilic aerobe of the *Thermus/Deinococcus* group, which constitutes an ancient lineage related to Cyanobacteria. *In silico* analysis revealed the relationship between the presence of *nirS* genes and phytopathogenicity in Gram-negative bacteria.

## Introduction

Nitric oxide (NO) is a toxic compound, able to bind to proteins that contain heme, iron, or copper (Torres and Wilson, 1999), resulting in protein disruption. Additionally, NO reaction with reactive oxygen species (ROS), namely with superoxide (O_2_^-^), inside a host cell produces highly reactive nitrogen species (RNS), e.g., the oxidizing compounds peroxynitrite (ONOO^-^) and the free radical nitrogen dioxide (NO_2_). The first is a molecule reactive with all major classes of biomolecules, thence mediating cytotoxicity independently of NO or O_2_^-^, while NO_2_ initiates lipid oxidation (Patel et al., 1999). On the other hand, NO chemistry is highly relevant in cell signaling, e.g., peroxynitrite reaction may lead to nitrosation (addition of NO) or nitration (addition of NO_2_) with the formation of compounds (e.g., nitrotyrosine, *S*-nitrosoglutathione) that intervene in a variety of cell signaling pathways (Baudouin and Hancock, 2014).

Nitric oxide is currently considered a ubiquitous signal in biosystems, playing significant roles in the response to environmental stimulus (Baudouin and Hancock, 2014). This ubiquitous character is certainly related to its high chemical reactivity resulting in an elevated number of target molecules that may undergo changes in their structure and activity (Astier and Lindermayr, 2012). As a consequence, transformations of some of these target molecules may involve changes at gene transcription levels (Astier and Lindermayr, 2012). Although numerous NO target molecules have been identified, their large numbers hamper the knowledge on how NO signals translate into coordinated downstream effects and specific outputs. In particular, unveiling the NO response network involving biota interactions is a great challenge. For example, NO is highly relevant in the signaling involved in relationships between plant and microbes and within microbial communities. In these systems, the fundamental knowledge of NO-source, NO-sensing, and NO-dependent transduction remains mostly unknown. In any case, such NO-dependent relationships certainly lead to the occurrence of interactive adaptations during evolution, which were translated into unique intricate NO circuits. The reconstruction of these evolutionary adaptations may provide significant insights into the rationale of NO circuits.

Herein, we propose that local NO accumulation during the evolution from anaerobic to aerobic microbial lifestyles could have triggered phytopathogenesis and control other bacteria–plant interactions. Our streamline, described in the sections below, was based on preliminary assumptions. It is estimated that in early Earth, the production of NO was *ca* 10^13^ g per year (Wang et al., 1998). NO produced by lighting would have been converted into nitric (HNO_3_) and nitrous (HNO_2_) acids and transported as acid rain to the ancient ocean and early lithosphere. NO and nitrous-acid-derived nitrite might have been the electron acceptors of primitive biological denitrification pathways in the early Archean biosphere (*ca* 3.8–2.5 Gya) (Moroz and Kohn, 2011). Thus, denitrification in the Archean anoxic atmosphere, associated with NO bioavailability, may have been the basis for a long-term evolutive trend regarding NO biosource and signaling. Chen and Strous (2013) proposed a coevolution of denitrification and aerobic respiration pathways, supported by their dependence on the same core molecular machinery, and the experimental evidence for the corespiration of nitrate and oxygen (see ref therein). Chen and Strous (2013) also highlighted the advantage of this phenomenon in ecosystems with steep oxygen gradients, allowing the channeling of redox excess flow resulting from oxygen level fluctuation. An evolutionary history where terminal oxygen oxidoreductases developed from nitric and nitrous oxide reductases had previously been pointed out (Castresana and Saraste, 1995). Consequently, the coexistence of denitrifier/aerobe microbiota and oxygen-producing early Cyanobacteria in primitive microoxic habitats and the evolution of interactive adaptations between them can be envisaged. The expansion of aerobiosis followed the GOE (between *ca* 2.4–2.3 and 2.1–2.0 Gya) caused by cyanobacterial blooms. The increasing oxygen level could have resulted in the consequent inhibition of NO reductase and a NO surplus in the denitrification pathway (Feelisch and Martin, 1995). This accretion may have triggered phytopathogenesis when an oxygen-coping denitrifier would use NO to outcompete Cyanobacteria. Along with evolution, a multitude of sequential adaptive responses could eventually lead to an intricate web of biotic responses and translate into pathogen–host interactions that ultimately could result in disease or disease-like consequences.

Establishment of a plant disease involves complex interactions, as successful bacterial plant pathogens must detect hosts and circumvent their defenses. Plant pathogens developed during evolution a tuned capacity for sensing and responding to environmental and plant stimuli, exerted by the joint action of multiple crosstalk effectors. However, NO surplus, as in primitive biota interactions, represents a major determinant in the outcome of biotic relationships. At present, pathogen-derived NO is an important molecule involved in virulence and survival in the plant host (Arasimowicz-Jelonek and Floryszak-Wieczorek, 2014). Thus, inspection of the phylogeny for NO-forming nitrite reductases could allow the identification of key genes and hidden features related to phytopathogenesis. Herein, a potential key ancestor of nitrite reductase gene, *nirS*, has been identified in *Thermus thermophilus*, a member of an ancient phylum with a close evolutionary link to Cyanobacteria. We propose the *nirS* gene may represent a target for the preliminary diagnosis of potential phytopathogens within Gram-negative bacteria.

## NO Biosynthesis Pathways

**FIGURE 1:**
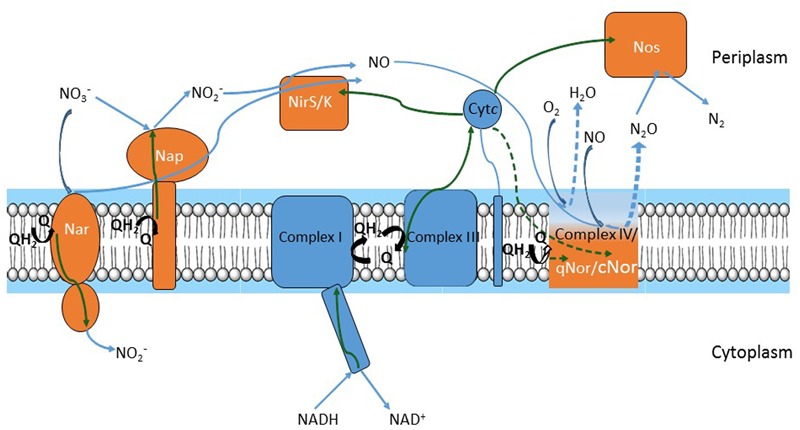
Respiratory chains of denitrification and oxygen reduction. Electron flow is represented by green arrows; substrate flow is represented by blue arrows. Electrons move from NADH to nitrite reductases (NirS or NirK) and nitric oxide reductases (qNor or cNor) or to oxygen oxidoreductase (Complex IV) via Complex I, quinone/quinol pool, Complex III and cytochrome *c.* Alternative pathways in electron flow (i.e., cytochrome *c* electron flow to Complex IV and cNor versus quinone flow to qNOr) or in substrate (i.e., oxygen reduction in Complex IV versus nitric oxide (NO) reduction by qNor or cNor) are depicted by dashed green and blue arrows, respectively. Complexes of the oxygen respiratory pathway are drawn in blue and those of the denitrification pathway in orange, except for Complex IV, qNor and cNor, which are in gradient color and represented by a unique module to point out their common evolutionary history.

Nitric oxide (NO) is an intermediate in the process of bacterial denitrification. A membrane-bound nitrate reductase (Nar) or a periplasmic nitrate reductase (Nap) reduces incoming nitrate (NO_3_^-^) to nitrite (NO_2_^-^). NO_2_^-^ produced in the cytoplasm is secreted and reduced to NO either by a heme (NirS)- or copper (NirK)-containing periplasmic nitrite reductase. Noteworthy, NirK sequences are highly diverse among bacteria (Decleyre et al., 2016). NO is immediately reduced to less toxic nitrous oxide (N_2_O) by cytochrome *c* (cNOR)- or quinone (qNOR) membrane-bound reductases. Complete denitrifiers have a periplasmic reductase (NosZ) which reduces N_2_O to nitrogen (N_2_) (**Figure 1**). According to the NOx reductase diversity, nine denitrification gene clusters were identified in a sequence-based screening from a soil metagenomic library (Demanèche et al., 2009).

Denitrification and aerobic respiration (the electron transfer pathway for oxygen reduction both in prokaryotes and in mitochondria) share common elements (Chen and Strous, 2013). A link between oxygen and NO reduction has been previously suggested, as nitric oxide reductases (NORs) are distant homologs of oxygen oxidoreductases (Castresana and Saraste, 1995; Zumft, 2005). Core respiratory elements – NADH dehydrogenase (Complex I), the quinone pool, the *bc*_1_ complex (Complex III), and cytochrome *c* – sustain both denitrification and aerobic respiration pathways. Each of the two pathways adds specific components to this template (**Figure 1**): aerobic oxidation requires a cytochrome *c-* or quinol-terminal oxygen oxidoreductase (Complex IV). Denitrification consists of the NOx reductases; denitrifiers contain at least two or three of these enzymes and produce N_2_O or N_2_ gas. To our knowledge, all denitrifiers are also able to perform aerobic respiration, thus a branched respiratory chain can be visualized (**Figure 1**). Denitrification steps proceed sequentially in the electron transfer from NO_3_^-^ to N_2_ but operate in parallel considering the core machinery, because denitrification and oxygen reduction components accept electrons from Complex I, cytochrome *c*, or the quinol pool.

Other pathways can also result in NO biosynthesis. Ammonia-oxidizing bacteria produce NO and N_2_O under transition between anoxic and oxic environments and/or excessive ammonia loading. The biosynthesis couples ammonia oxidation and denitrification (Chandran et al., 2011).

Nitric oxide synthases (NOS) catalyze NO production from the oxidation of L-arginine to L-citrulline, the presence of oxygen is obligatory for NOS-catalyzed NO production. NOS are present in all kingdoms, and multiple gene and genome duplication events, together with changes in protein architecture, resulted in NOS divergent isoforms (Andreakis et al., 2011). In mammals, there are three distinct NOS isoforms; they consist of homodimers, each monomer has an N-terminal oxygenase domain and a C-terminal flavoprotein reductase domain (Alderton et al., 2001). Prokaryotic NOS have been identified mainly in Gram-positive bacteria (bNOS) and Archaea (Wang and Ruby, 2011) and lack the reductase domain. Nevertheless, they use redundant reductases as electron donors to produce NO *in vivo* (Gusarov et al., 2008).

While there is no doubt of the relevance of NO presence in plants, where it has important roles in plant physiology processes (Baudouin and Hancock, 2014 and ref therein), no consensus exists about its generation pathway. NOS-like activity is considered the most important source of NO accumulation in plants (Groß et al., 2013). However, the existence of NOS enzymes in plants is under debate as no evidence of NOS sequences in sequenced plant genomes has been found and the robustness of many of the inhibitors and probes used in the search of NOS is questioned (Hancock, 2012). Contrarily, assimilatory nitrate reductase has long been known as a source for NO production. The enzyme reduces NO_3_^-^ to NO_2_^-^ at the expense of NAD(P)H, but may also catalyze an electron transfer from NAD(P)H to NO_2_^-^ resulting in NO formation. This NO producing capacity, several times overestimated, has been shown to represent about 1% of the nitrate reduction capacity (Planchet et al., 2005). The presence of a root-specific, plasma membrane-bound nitrate reductase, associated with a nitrite:NO oxidoreductase, which catalyzes the reduction of apoplastic NO_2_^-^ into NO, has been reported (Stöhr et al., 2001). Because the rate of NO production depends on the availability of nitrate and oxygen, the function of these enzymes in a “plant–root denitrification” process was considered. It was proposed that the NO produced may be an indicator of the external nitrate availability under anoxia and a regulator of symbiotic interactions at the root surface (Stöhr and Stremlau, 2006). Moreover, plant mitochondria, as well as those from animals, also produce NO under anoxia. In both cases, a nitrite:NO reductase activity, associated with cytochrome *c* oxidase, is blocked by inhibitors of respiratory electron transport (Planchet et al., 2005; Lacza et al., 2009). It has been suggested that nitrite is a reserve of NO, being reduced by the mitochondrial electron transport system to bioavailable NO during hypoxia to mediate physiological responses (Gladwin et al., 2005). By reducing NO_2_^-^ to NO, mitochondria also preserve the capacity to oxidize external NAD(P)H and maintain a limited ATP synthesis under anoxia. When oxygen concentration increases, NO participates in the mitochondria O_2_ homeostasis by competitively inhibiting cytochrome *c* oxidase which can oxidize it to NO_2_^-^ (Gupta and Igamberdiev, 2011). Apart from the potential physiological relevance of these regulatory mechanisms, the subsistence of these reactions reinforces the concept of a common evolutionary history for denitrification and aerobic respiration. It also follows that nitrogen oxides/oxygen availability have a role in NO plant level balance, which must be regulated considering the exposure of plants to NO from external sources and the scavenging activity and detoxification for NO within the plant tissue. Surely, nitrogen oxides derived from microbial activity have an important impact regarding the exposure of plant tissues to NO, triggering adequate plant responses, which involve, after all, mechanisms similar to those induced by microbial pathogens.

## The Dualism of Nitric Oxide

Nitric oxide (NO) produced by plant growth promoting rhizobacteria (PGPR), which enhance plant growth by direct and indirect mechanisms, is an intermediary in auxin-regulated signaling cascades influencing root growth and developmental processes, namely the induction of adventitious root formation (Molina-Favero et al., 2007). On the other hand, NO is implicated in the virulence of bacterial pathogens, with evidence for the role of microbial generated NO in the activation/deactivation of phytotoxins (Wach et al., 2005) and in the defense against immune oxidative burst. During this burst, the plant production of microbicidal ROS O_2_^-^ and H_2_O_2_ (hydrogen peroxide) is antagonized through NO-mediated activation of bacterial catalase and suppression of the damaging effects of the Fenton’s reaction (Shatalin et al., 2008). The importance of NO in plant resistance has been well documented, and studies have shown that a NO burst in plant cells after pathogen attack functions as a messenger in gene defense responses (Schlicht and Kombrink, 2013). Thus, NO is a ubiquitous molecule that can favor both the invader and the host. Yamasaki (2004, 2005) proposed an evolution concept for this dual relationship: initially the invader could produce harmful molecules (e.g., NO and derived reactive nitrogen oxide species). Later, defense responses could have been generated and evolved to mechanisms of tolerance and new roles for NO. This dualism of NO, (i.e., harmful and beneficial effects) suggests pathogens could have been one of the earliest steps on the evolutive history of interactive adaptation of microorganisms and plants. Thus, originally, NO could have been a signaling molecule whose role was to limit photosynthetic microorganisms’ development, which has led to conquer host “plants.”

It is important to point out the analogy between NO and ROS dualistic behavior. ROS are toxic by-products of energy metabolism, causing lipid peroxidation and membrane damage (Montillet et al., 2005), however, they are also signals that modulate development and stress responses. For instance, ROS have been implicated in the plant root architecture formation (Liu et al., 2016) and in the activation of plant stress-response pathways to abiotic and biotic stress (Levine et al., 1994; Groß et al., 2013). On the contrary, some pathogens may synthesize ROS in the infected tissue to induce cell death that facilitates subsequent infection (Govrin and Levine, 2009). Moreover, an intricate interplay has been tuned during evolution between NO, ROS, antioxidants (e.g., glutathione), and antioxidant enzymes (reviewed by Groß et al., 2013). For instance, H_2_O_2_ or NO treatments indicate that NO may act as a direct ROS scavenger (see below) and an inducer of the antioxidant system (Groß et al., 2013). As examples, salt stress in plants, similar to other stress, results in accumulation of ROS, which cause lipid peroxidation (You and Chan, 2015). Malondialdehyde (MDA) is a by-product of this process, and thereby an indicator of the extent of damage of plant membranes. In barley, treatment with a NO donor alleviated the damage of salt stress, namely the level of lipid peroxidation, and increased the activities of antioxidant enzymes superoxide dismutase (SOD), ascorbate peroxidase (APX), and catalase (CAT), suggesting a protective role of NO by enhancing the activity of these enzymes to suppress excessive ROS (Li et al., 2008). Mostofa et al. (2015) reported that H_2_O_2_ treatment prior to rice seedlings exposure to salt stress lowered ROS accumulation and the level of the MDA stress marker, and enhanced APX and CAT activity compared to the nontreated salt-stressed seedlings. However, the pretreatment decreased SOD activity under salt stress. With no pretreatment, a higher SOD activity under salt stress increased the conversion of O_2_^-^ to H_2_O_2_ but increased H_2_O_2_ mediated oxidative damage due to a nonmatching CAT activity. The authors proposed that in addition to the increased CAT activity, endogenous NO, whose content was elevated following H_2_O_2_ pretreatment, scavenged O_2_^-^directly. Adding a NO scavenger abolished the beneficial effects of H_2_O_2_, supporting the notion that NO had a major role in reducing ROS and MDA through its scavenging properties, and signaling role for the increase of antioxidant enzymes activity. In agreement, improved stress tolerance concomitant with a decrease in H_2_O_2_ and MDA levels is a general result reported for exogenous NO treatments (Groß et al., 2013). NO treatments could thus induce a primed state preparing plants to efficiently respond to future stress episodes (Conrath, 2011). Contrarily, NO donors have been reported to be associated with the inhibition of antioxidant enzymes APX and CAT in tobacco (Clark et al., 2000).

The involvement of NO both in increased and in decreased antioxidant enzyme activities and ROS levels may be explained if a NO dose-dependent effect on the cellular redox status is considered (Thomas et al., 2008). At low concentrations, NO reactions with organic molecules and oxygen species would translate in NO-induced cell signaling, stimulating the antioxidant stress responses while high NO concentrations would boost nitrogen oxide reactions inflicting severe cell damage. This model is well suited to explain the phytopathogen/plant host initial interaction linked to the plant immune oxidative burst cited above. Following recognition of a pathogen, a plant hypersensitive response (HR) is triggered, and the pathogen stimulation of apoplastic ROS via consumption of oxygen, the oxidative burst, is one of the earliest plant–cell responses that ultimately leads to rapid cell death at the infection site, preventing the spread of infection (Torres et al., 2006). It was demonstrated that NO is another essential messenger in HR cell death; ROS and NO donors trigger cell death most efficiently in conjunction (Delledonne et al., 1998) suggesting cooperation in cell death signaling. Besides, uncontrolled amplification of ROS/RNS signaling might provoke nitrosative stress and ultimately irreversible cell damage.

In summary, NO (and ROS) dualistic nature regards their ability in signaling either the promotion of stress defense or pathogenesis. The NO level is determinant for pathogenesis, an ability for a high NO level synthesis from the pathogen might stimulate disease, whereas a lower level synthesis by a PGPR might be determinant in plant development process, for instance in root growth (Molina-Favero et al., 2007). The importance of the NO level in an evolutionary background is described in the sections below, where possible chronological events leading to the actual intricate NO–ROS interplay and NO signaling are presented. In particular, NO and ROS (and RNS) close association might be further comprehended considering a common evolutionary history for NO accumulation and ROS synthesis.

## Nitric Oxide: An Ancient Messenger

Comparative data on the physiology and biochemistry of NO-mediated pathways, widespread across domains, suggests deep phylogenetic roots for NO gaseous signaling and point out that “NO-coupled regulatory systems may be as old as cellular organization itself,” placing their origin at the morn of biological evolution *ca* 3.8–3.5 Gya (Moroz and Kohn, 2011).

Under the anoxic primitive Earth, geological and atmospheric conditions were in favor of NO synthesis and accumulation. Several authors have underlined the early chemical synthesis of NO in the primitive Earth’s atmosphere and oceans during the Hadean and Archean eras (from 4.5 to 2.5 Gya) by volcanism and lightning (Nna Mvondo et al., 2001; Martin et al., 2007). Synthesis of NO has been suggested as a crucial factor to the origin of life itself. Nitrogen is essential for life; however, most organisms are not able to use atmospheric N_2_, since a high energy is required to dissociate the N_2_ triple bond. Available nitrogen for organisms should be as reduced (e.g., ammonia: NH_3_/NH_4_^+^) or oxidized (N_2_O, NO, NO_2_^-^, or NO_3_^-^) forms and NO was proposed as one of the major sources of utilizable nitrogen in the primitive Earth.

NO would have been converted into nitric and nitrous acids, transported to early lithosphere and ancient ocean as acid rain. NO and related species might have been reduced to ammonium (NH_4_^+^) by ferrous iron, present in ancient oceans, possibly leading to zones of NH_4_^+^accumulation for the synthesis of amino acids and other biochemicals. Additionally, NO and nitrites might have been the electron acceptors for the development and evolution of ancient cellular respiratory machinery representing the primitive biological denitrification pathways in the Archean biosphere (*ca* 3.8–2.5 Gya) (Moroz and Kohn, 2011 and ref therein).

At *ca* 2.4–2.3 Gya, oxygen concentration in Earth’s atmosphere rose, triggering the development of novel biotic adaptive responses to an oxygen rich atmosphere, namely the expansion of aerobic respiration. Possibly, these early responses coexisted with ancient roles of NO expressed under hypoxia, leading further to joint adaptive responses resulting from antioxidant properties of NO. In fact, NO can recede Fenton-mediated oxidative stress by direct scavenging of hydroxyl radicals formed in the reactions between H_2_O_2_ and transition metals, and terminate lipid peroxidation by reaction with peroxy and oxy radicals (Wink et al., 2004). Moreover, as mentioned above, NO scavenges O_2_^-^ to give ONOO^-^, a short-lived molecule in the cell, which, despite being itself an oxidant, mediates important cell-signaling process (Groß et al., 2013). Hence, NO chemistry under oxic conditions might have provoked the evolution of important regulatory and coupling mechanisms, where it both modified and subsequently acted as a messenger of cell oxidative status.

## Relevance of Thermo-Adaptation Through Earth Evolution

The accumulation of free oxygen in the early Earth’s atmosphere is known as the GOE, also called the Oxygen Catastrophe. Cyanobacteria, which appeared *ca* 200 million years before the GOE, were the inductors of this phenomenon, because cyanobacterial photosynthesis resulted in the accumulation of free oxygen in the atmosphere. A consequence of the GOE was the lowering of planet temperature; there is evidence that the Earth’s earliest ice age was due to the GOE (Tang and Chen, 2013). Thermo-adaptation must have been determinant for life maintenance under those conditions. Low temperatures reduce molecular movement diminishing enzyme activity and other dynamic cell functions. Accordingly, mesophiles have several modifications, compared to thermophiles, to respond to decreased molecular motion, as for instance more fluid membranes with more unsaturated lipids, proteins with reduced hydrophobic cores and higher substrate affinity (López-García et al., 2015). These modifications involved changes in the amino acid composition of proteins and consequently in codon bias; thus, temperature adaptation integrated in the genome of these cells and other potential temperature-related physiological adaptive mechanisms could have been developed (Cuecas et al., 2016). In this context, it has been demonstrated that optimal growth temperature is a major factor affecting the patterns of codon usage among prokaryote genomes (Lynn et al., 2002).

## Horizontal Gene Transfer and the Colonization of Low-Temperature Environments

**FIGURE 2:**
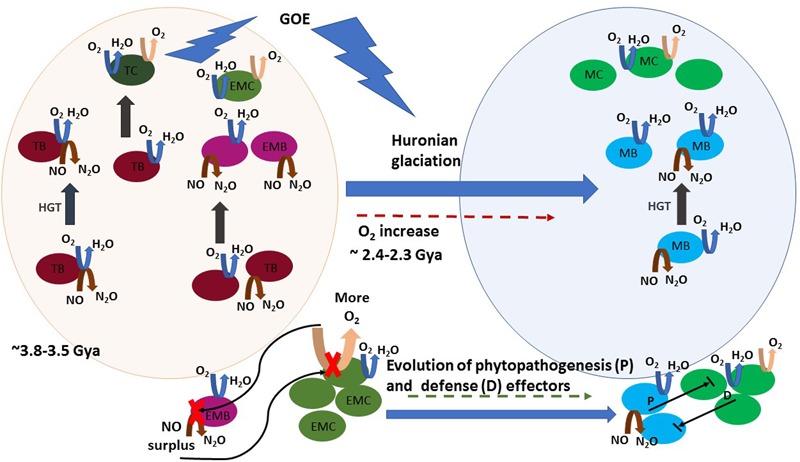
A schematic view of the proposed hypothesis on the evolutionary history of NO production and phytopathogenesis. Horizontal gene transfer (HGT) is represented by gray straight arrows. Denitrification, aerobic respiration, and photosynthesis reactions are represented by brown, blue, and pink curved arrows, respectively. Round shapes represent bacteria: thermophilic bacteria (TB, dark pink), thermophilic Cyanobacteria (TC, dark green), early mesophilic bacteria (EMB, pink) early mesophilic Cyanobacteria (EMC, olive green), mesophilic bacteria (MB, blue), and mesophilic Cyanobacteria (MC, green). A timeline is represented from early life conditions (*ca* 3.5 Gya), when a primitive atmosphere was essentially anoxic or microoxic (represented by the light pink sphere) to the development of an oxic atmosphere period, where facultative anaerobe and aerobe lifestyle is abundant (represented by the light blue sphere). Initially, HGT spread an ambivalent NO/oxygen reduction gene cluster (represented by the coincident blue and brown arrows in TB) in the early microbiota, which was subjected to speciation to oxygen reduction. Later, HGT of independently evolved aerobic respiration or denitrification gene clusters proceeded among thermophilic and early mesophilic bacteria, the latter arose in microoxic niches before the GOE. Note that these HGT events were probably widespread between intra- and inter-taxonomic groups of evolving thermophilic and mesophilic bacteria, and they are not all depicted. Atmospheric oxygen rose during GOE, due to cyanobacterial blooms, and led to the increase of aerobic mesophilic lifestyle. The oxygen boost inhibited nitric oxide reductase (NOR) in early microaerophilic and anaerobe denitrifiers and caused NO accumulation. NO toxicity caused “the first disease” in Cyanobacteria. During evolution both evolving Cyanobacteria and phytopathogens developed numerous defense and phytopathogenesis effectors, respectively. Establishment of the “disease” results from the balance between the effectors’ activity of both intervenient and the eventual NO surplus production by one of them is still a major determinant in the outcome of their interaction.

At lower temperatures, oxygen dissolves more efficiently and becomes easily accessible, causing heterotrophy based on aerobic respiration, an increasingly dominant trait of mesophilic lifestyle. The acquisition of genes for aerobic respiration certainly seems to have been determinant in the ability to colonize vast low-temperature environments. As aforementioned, denitrifying and oxygen-reducing respiratory chains have common features and the putative evolution of an ancient terminal oxidase that reduced both oxygen and NO, as it is nowadays observed for heme–copper oxidases of *T. thermophilus* (Giuffre et al., 1999), has been proposed (Brochier-Armanet et al., 2009; Chen and Strous, 2013). The simplest respiratory chain consisted of a form of Complex I (NADH dehydrogenase) that reduced a quinone followed by its reoxidation by a terminal oxidase, a Complex IV (a quinol NO/oxygen reductase) (Chen and Strous, 2013). Thus, thermophilic bacteria adapting to a mesophilic lifestyle were possibly also adapting primordial biological denitrification pathways to oxygen respiration. Both systems, denitrification and aerobic respiration, could coexist and manifest as a function of the local presence of anoxic or oxic conditions. In that process, horizontal gene transfer (HGT) may have been crucial. Indeed, comparative analysis of thermophilic bacterial genomes with those of other phylogenetically related mesophilic bacteria showed that insertion sequences are shared between thermophiles and mesophiles (Takami et al., 2004). Brochier-Armanet et al. (2009) investigated the distribution and phylogeny of the catalytic subunits of different types of oxygen terminal oxidases and proposed different evolutionary histories involving HGT. The heme–copper terminal oxidases of the A family, which comprises the mitochondrial terminal oxygen reductase, is characterized by the presence of D- and K-proton channels in the catalytic subunit I. They form a consistent group in terms of amino acid similarities (Pereira et al., 2001) and have been suggested to be the most ancient type of oxygen oxidoreductases. These oxidases likely originated prior to the emergence of oxygenic photosynthesis in the cyanobacterial lineage. This suggested that the ability for oxygen reduction was not a consequence of the increased oxygen production by Cyanobacteria but the possibility of divergence of pathways in early microbial life in the presence of low quantities of oxygen in Archean atmosphere. Targeted metagenomics indicated that the gene clusters involved in denitrification were also probably subject to shuffling by HGT between bacteria (Demanèche et al., 2009). The puzzled presence of the denitrification genes in different related strains is an example of the known plasticity of bacterial genomes (Cuecas et al., 2017). An example is the presence of a denitrification genomic island which can be spread by HGT among *T. thermophilus* strains (Alvarez et al., 2011; Bricio et al., 2011). In summary, denitrification gene sequences evolving to an oxygen respiration pathway were likely horizontally transferred during the GOE and subjected to positive selection, along with thermo-adaptation traits, in the course of the increased oxygenation occurring in that period (**Figure 2**).

## NO Accumulation and the Establishment of Antagonistic Biotic Associations

The increased oxygenation during GOE caused NO bioaccumulation, as the transition from globally anaerobic to aerobic conditions could competitively inhibit NOR activity during denitrification (Feelisch and Martin, 1995). It is indeed well known that NO and oxygen reductases show cross reactivity with respective substrates (Fujiwara and Fukumori, 1996; Giuffre et al., 1999; Duarte et al., 2014). NO accumulation could be a pressure for the selection of microbes able to reduce oxygen, because this reduction ability would be used for scavenging oxygen in an anoxic habitat. Thus, gene-encoded structural modifications of NOR to utilize oxygen as substrate, and further spreading out by HGT within an initial population, might have been selected in transient oxic niches. In this scenario, one can conceive that an early denitrifying prokaryotic cell with machinery adapted to cope with aerobic conditions, would benefit from a transient NO surplus as a signal to inhibit oxygen production by Cyanobacteria in an oxic niche – NO is known to slow down photosynthetic electron transfer (Wodala et al., 2008). Additionally, in oxygen-enriched aqueous solutions, NO is primarily oxidized to NO_2_^-^ (Ignarro et al., 1993), and this behavior could be a benefit to denitrifiers in an aerobic niche.

Noteworthy, NO accumulation could also have triggered ROS accumulation by an indirect mechanism. The NO surplus, caused by the inhibition of a primitive ambivalent NO/oxygen reductase by oxygen, might have been associated with a deregulation of the electron flow in the denitrification/aerobic pathway. As the reduction ability of NOR was lower for oxygen than for NO, this would cause the accumulation of electrons in the respiratory chain complexes ahead. Supporting this view, is the fact that, in mitochondria, O_2_^-^ production increases as the respiratory chain becomes more reduced (Turrens, 2003). Complex I, above cited as a shared element in the denitrification/aerobic respiratory chain, is a major locus for ROS generation; the complex catalyzes both O_2_^-^ and H_2_O_2_ formation in the presence of NADH (Grivennikova and Vinogradov, 2013). Additionally, transient inhibition of cytochrome-*c* oxidase by NO enhances O_2_^-^ production (Moncada and Erusalimsky, 2002); this inhibition could be relevant for a population of denitrifier facultative aerobes searching to outcompete other microbes in an oxic niche.

The proximity with Cyanobacteria in the oxic niche could develop to a tolerance and even further to a mutualism, when a heterotrophic denitrifier facultative aerobe comes to profit from cyanobacterial organic metabolites (i.e., carbohydrates). The evolutionary outcome of these interactions would depend on both the interacting members and the external factors. Consequently, a unique NO circuit could be the basis for the establishment of either phytopathogenesis or beneficial plant–microbe interactions. Thus, from its putative origin, defining a microbe as mutualist or pathogen must cross a thin and ambiguous line, above all, after new data are revealing reversions and transitions between beneficial and detrimental associations (Pérez-Brocal et al., 2013).

## NO Level: A Determinant of the Outcome of Biotic Interactions

If a unique NO circuit was the basis for the establishment of a biotic interaction, the outcome as a hostile versus mutualistic association should have been the result of an equilibrium between the NO producing and detoxifying ability of the partners; a phytopathogen would be the microbe with the highest NO producing rate and highest tolerance to NO and presenting the ability to cope with oxygen. During evolution, alternative denitrification and other NO synthesis pathways have been developed. For instance, in the L-arginine-NO pathway, NOS use oxygen; hence, this pathway might have been the result of adaptive responses to a post-GOE oxygen-rich atmosphere (Moroz and Kohn, 2011). bNOS have been implicated in the virulence of *Streptomyces* and *Bacillus* (Wach et al., 2005; Shatalin et al., 2008), but genes encoding bNOS also exist in the genomes of nonpathogenic soil bacteria (Gusarov et al., 2009). Also, other effecters were acquired, lost, or modified to enhance competitive advantages as, for instance, antibiotic production, Type III (T3SS or injectisome), and type VI (T6SS) protein secretion systems (used by Gram-negative bacteria to sense the host and to secrete effector proteins that target immune signaling system (Macho and Zipfel, 2015) or cell integrity (Alteri and Mobley, 2016), respectively) and genome reduction in obligate pathogens and symbionts (Moran, 2002). There are numerous reports on genomic comparisons between related (phyto)pathogenic, nonpathogenic, symbiotic, and PGPR strains based on the presence and number of protein-encoding genes classified as beneficial or virulent. Bruto et al. (2014) targeted 23 genes known to have a role in established PGPR effects from the genome sequences of 304 Alpha-, Beta-, and Gammaproteobacteria. They found the number of genes contributing to plant-beneficial functions increased along the continuum animal pathogens, phytopathogens, saprophytes, endophytes/symbionts, and PGPR, and suggested that “the accumulation of these genes (and possibly of different plant-beneficial traits) might be an intrinsic PGPR feature.” Cesbron et al. (2015) used also comparative genomics to detect differences between *Xanthomonas arboricola* strains; pathogenic strains possessed a larger number and variety of mobile genetic elements than nonpathogenic strains. The type III effector repertoire was larger in pathogenic strains and the sets of genes encoding chemoreceptors and adhesins were also different. Thus, numerous intricate factors are implicated in biotic antagonisms. Despite this, NO can still be a determinant compound in the outcome: for example, NO inhibits the transcription of *Salmonella* pathogenicity island-2 Type III secretion system (McCollister et al., 2005) and modulates the antimicrobial activity of antibiotics (Gusarov et al., 2009; Jones-Carson et al., 2014). Thus, research on pathogenesis based on comparative studies should consider the presence, type, and number of systems for NO production and detoxification. Our hypothesis suggests that phytopathogens maintain a fast pathway for NO synthesis, that could be a partial signature of their active role in phytopathogenesis. Since denitrification and oxygen respiration showed paralleled evolution paths, the latter a major process following photosynthesis evolution and Cyanobacteria bloom (**Figure 2**), we propose that denitrification genes may constitute such signature. Recently, Dalsing et al. (2015) have shown that the phytopathogen *Ralstonia solanacearum* uses its denitrification pathway to benefit from host nitrate to grow and cause disease, and mutant strains lacking nitrite reductase showed reduced virulence. Also, D’Amico and Filiatrault (2017) demonstrated that a hypothetical protein of unknown function – PSPTO_3957 – was necessary for nitrate assimilation and full virulence in the plant pathogen *Pseudomonas syringae*. However, NO production via the denitrification pathway during pathogenic processes on plants has been poorly characterized and such deficit should be filled.

## NirS From *Thermus thermophilus*: An Ancient Signature of NO Synthesis

The genus *Thermus* of the *Thermus/Deinococcus* Phylum is part of one of the oldest phylogenetic groups of the Bacteria Domain (Wu et al., 2009). The cell envelope of *T. thermophilus* is a complex pattern of layers with properties intermediate between those of Gram-positive and Proteobacteria (Acosta et al., 2012). Moreover, the *Thermus/Deinococcus* group and Cyanobacteria constitute deep-branching divisions specifically related to each other and these two groups have been reported to branch off from early ancestors of Gram-negative bacteria (Sarma, 2012). Noteworthy, the A family terminal oxidases from *T. thermophilus* and Cyanobacteria have all the residues of the D- and K-channels except the glutamate (GluI-278) at the hydrophobic end of the D-channel, a key residue for proton conduction. Instead, they use two consecutive residues – YS – in proton transfer, as it is the case of other members of the A family, which are therefore classed to A2 subfamily (Pereira et al., 2001). The presence of A2 subfamily in *Thermus* and Cyanobacteria old phylogenetic bacterial groups as well as the ability of heme–copper terminal oxygen reductases from *T. thermophilus* to catalyze the reduction of NO to N_2_O under anaerobic conditions (Giuffre et al., 1999) are arguments to envisage a shared evolutionary ecology history for these lineages, i.e., the interaction of their ancestors in a niche where the microaerophilic or aerotolerant lifestyle dominated.

In *T. thermophilus*, a nor–nir supercluster can be spread by HGT among different strains (Alvarez et al., 2011). These nor and nir sequences encode a cNOR reductase (Schurig-Briccio et al., 2013) and a cytochrome *cd*_1_ type NirS reductase. Considering the ancient *Thermus* lineage, the denitrification/oxygen respiration evolution, and the phylogenetic relationship between *Thermus/Deinococcus* group and Cyanobacteria, it is herein postulated that the presence of a NirS reductase homologous to the NirS from *T. thermophilus* could constitute an ancient signature related to NO synthesis with relevance to antagonistic biotic associations, for example, in phytopathogenic Proteobacteria.

## Phytopathogenesis and the Distribution of NirS in Bacteria

A Blast analysis was performed using the Blast tool (National Center for Biotechnology Information, United States) targeting relevant taxonomic genera from Gram-positive and negative bacteria known to include phytopathogens. Gram-positive bacteria comprised the following genera: *Clavibacter, Curtobacterium, Rathayibacter, Leifsonia, Nocardia, Rhodococcus, Streptomyces, Bacillus, Clostridium, Spiroplasma*, and *Candidatus Phytoplasma*; Gram-negative included *Agrobacterium, Sphingomonas, Candidatus Liberibacter* (α-Proteobacteria), *Acidovorax, Burkholderia, Ralstonia, Xylophilus* (β-Proteobacteria), *Erwinia, Pseudomonas, Xanthomonas*, and *Xylella* (γ-Proteobacteria). The Blast search was also made within the Rhizobiales order comprising nitrogen-fixing, legume-nodulating symbiotic bacteria.

**FIGURE 3:**
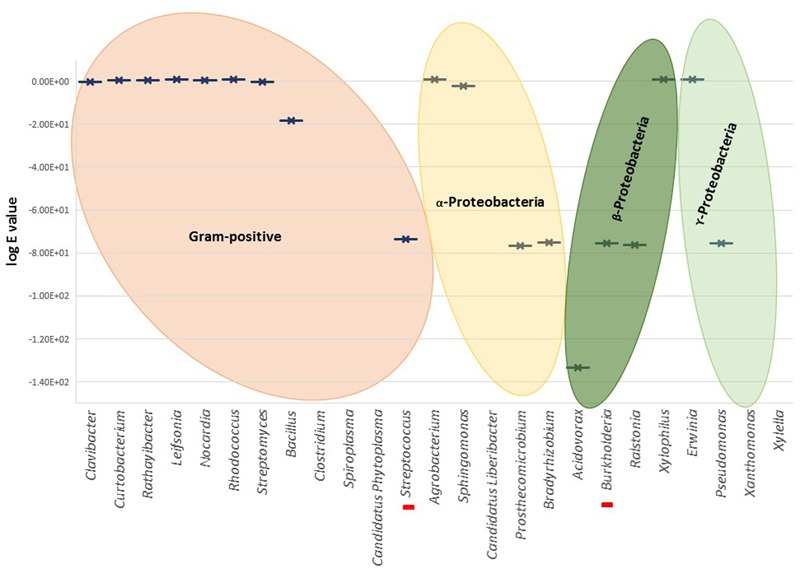
Graphical representation of the highest Blastp scores (log e value) for different genera. Red bars in the *X*-axis represent unique cases inside the represented genus, i.e., *Streptococcus pneumoniae* and *Burkholderia cepacia*. There is no graphical representation for the genera in *X*-axis without significant similarities found by Blastp.

No significant homologies were detected for *T. thermophilus* NirS in the Gram-positive genera (**Figure 3**). Among all Gram-positive, only *Streptococcus pneumoniae* showed a significant score (Blastp score 2e^-171^). The absence of related *nirS* genes in this large bacterial group could be partly explained by the fact that research has been dominated by molecular analyses based on genes from Gram-negative bacteria, and Gram-positive denitrifiers were neglected in the last decades (Verbaendert et al., 2011). Thus, distinct poorly known divergent *nirS* genes may be part of the denitrification process in Gram-positive bacteria; their presence is suggested by Blastp reported partial sequences of cytochrome *cd*1 nitrite reductase in several *Bacillus*.

Nitrite reductase NirS was found in Gram-negative Burkholderiales and Pseudomonales (i.e., genera *Acidovorax, Burkholderia, Ralstonia*, and *Pseudomonas*). From Blastp analysis within *Burkholderia*, only *B. cepacia*, a well-characterized pathogen of onion and banana and opportunistic pathogen in patients with cystic fibrosis, showed high homology to NirS (Blastp score 3e^-175^).

**FIGURE 4:**
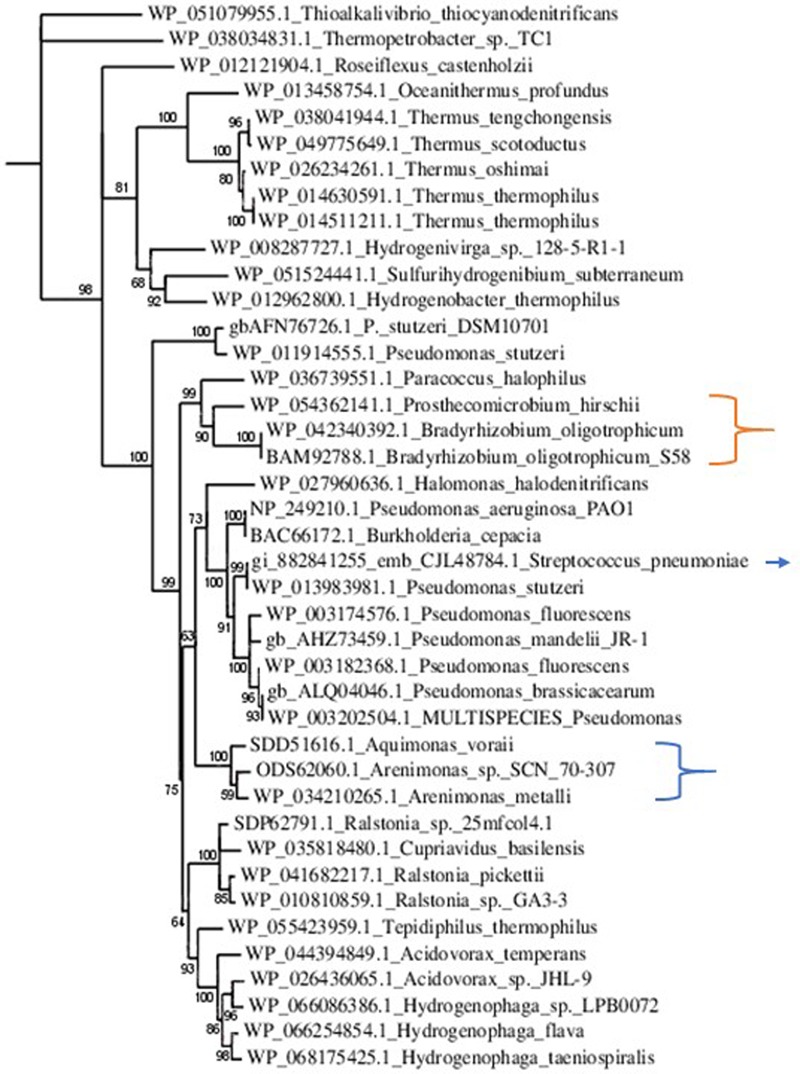
Phylogenetic tree of NirS sequences. Sequences were aligned with MUSCLE (v3.8.31) and ambiguous regions were removed with Gblocks (v0.91b). The phylogenetic tree was reconstructed using the maximum likelihood method implemented in the PhyML program (v3.1/3.0 aLRT). The substitution model considers that the data set does not contain invariable sites and assumes a 4-gamma-distributed rate category to account for rate heterogeneity across sites. The gamma shape parameter was estimated directly from the data (gamma = 0.998). Reliability for internal branch was assessed using the aLRT test (SH-Like). Graphical representation and edition of the phylogenetic tree were performed with TreeDyn (v198.3). Branches with a support value smaller than 50% were collapsed. The Gram-positive *Streptococcus pneumoniae* is indicated with an arrow. It is part of a cluster comprising Gram-negative phytopathogens and opportunistic pathogens. This result suggests the acquisition of *nirS* by horizontal gene transfer. The Rhizobiales cluster is indicated with an orange bracket. Although there were no homologs to NirS in *Xanthomonas* and *Xylella*, that is not the case for other Xanthomonadales, which are herein pointed out by the blue bracket.

NirS was poorly represented within the Rhizobiales, solely *Prosthecomicrobium hirschii* and *Bradyrhizobium oligotrophicum* showed relevant Blastp scores. However, alignment and tree construction (**Figure 4**) revealed that the Rhizobiales formed an independent cluster, indicating an evolutionary divergence and possible functional distinction. Although *Bradyrhizobium oligotrophicum* has been described as a nitrogen-fixing symbiont of the aquatic legume plant *Aeschynomene indica* (Okubo et al., 2013), it is known that Rhizobia and phytopathogenic bacteria have common infection strategies, thus the symbiosis output of this strain might need to be further evaluated by inspecting the molecular basis of the symbiotic process (Okazaki et al., 2016).

At present, the presence of NirS per se is not a determinant of phytopathogenesis because some Gram-negative phytopathogenic strains do not possess a related NirS; other determinants of phytopathogenesis, including other class of nitrite reductases, e.g., the copper nitrite reductase in the agent of bacterial wilt disease in tomato *– R. solanacearum* – developed during evolution. Moreover, as aforementioned, defining a microbe as mutualist or pathogen in an exclusive way is unfeasible, the distinction between mutualism and pathogenesis is an oversimplification of a continuum, and the transition between these relationships will depend on specific gene content, gene interactions and regulation, and the environmental conditions. On the other hand, several nonphytopathogenic strains have NirS. However, in this case, many are biocontrol strains. For instance, most of the characterized NirS containing *Pseudomonas* strains are defined as biocontrol PGPR, whose presence antagonize pathogens, suggesting a potential agreement with the NO hypothesis herein presented. Many biocontrol strains are usually characterized by their dual antibiosis abilities and the tripartite interaction plant/pathogen/biocontrol strain has been scarcely studied at the molecular level, even though both pathogen and plant genes are modulated by the presence of the biocontrol bacterium (Daval et al., 2011). In this context, it is noteworthy to mention the work of Wang et al. (2005). Wang et al. (2005) reported that tomato plant infection with *R. solanacearum* was reduced in plants treated with *Pseudomonas fluorescens* NO-overproducing transformants compared with treatment with *Pseudomonas* wild type. The transformants were obtained by disruption of their NOR genes. These results suggest that NO production through denitrification using nitrite reductase is an important route involved in biocontrol relationships.

## Discussion and Perspectives

Despite our limited understanding of NO synthesis and regulation in denitrifiers, NO detoxification, NO full signaling potential, and nitrite reductases phylogeny and structure, we present evidence on the evolutive role of NO on bacteria–plant interactions and their putative consequences. NO synthesis by denitrification is proposed to be a primitive major source of NO as elicitor of antagonistic biotic relationships, namely of phytopathogenesis. A sequence with ancestral origin, *nirS*, encoding cytochrome *cd*_1_-type NirS reductase, retained in phytopathogenic Proteobacteria, could represent a potential target to be used as a diagnosis tool for the preliminary detection of phytopathogens within Gram-negative bacteria, and specifically in the orders Burkholderiales and Pseudomonales.

The results herein presented strongly point to the need for further research on NO biosynthesis and detoxification in denitrifiers and nitrite reductases homology and structure. Several questions arise and will drive further work, the first concerns on experimental evidence for our hypothesis: given a group with phytopathogens and nonpathogens close members, would the introduction of a *nirS* gene cluster in the latter be necessary and sufficient to convert them in disease agents to a specific host? How cocultures of *Thermus*/Cyanobacteria would respond to changes in NO content, e.g., how their relative abundances would fluctuate? Other related contents remain also to be explored, for instance: could NO reduction to N_2_O by terminal oxygen reductases be a wide mechanism in prokaryotes? Copper nitrite reductase NirK is spread in Gram-negative and Gram-positive bacteria and its sequence is highly diverse. Could a particular NirK sequence/structure be related to phytopathogenesis? Low Blast scores with NirS were detected for *Bacillus*. Are NirS-like sequences present in a large number of Gram-positive bacteria? Presence of a *nirS* homolog in *S. pneumoniae* suggests it was acquired by HGT from Gram-negative bacteria. Are there such *nirS* homologs in other Gram-positive? What are the adaptive modifications in NirS *S. pneumoniae* sequence and structure to function in Gram-positive bacteria?

Further research on the plant partner of the phytopathogenic interaction is needed. Clearly, the controversy regarding plant NO source must be placed in a larger perspective, one that takes in consideration the NO synthesis by plant-associated microbes. Possible synergies or antagonisms between NO synthesis/signaling between bacteria and the plant partner should be further explored using biochemical and molecular tools. We encourage researchers to experimentally approach these questions on NO and its relationship to microbe–plant interactions and phytopathogenesis.

## Author Contributions

MS draft the manuscript. JG and CC revised the manuscript critically for important intellectual content.

## Conflict of Interest Statement

The authors declare that the research was conducted in the absence of any commercial or financial relationships that could be construed as a potential conflict of interest.
